# ADAMTS-18 in the host tissues exerts little effect on breast tumor progress in a murine 4T1 breast cancer model

**DOI:** 10.1186/s12952-016-0045-0

**Published:** 2016-02-03

**Authors:** Min Liu, Tiantian Lu, Fang Jing, Suying Dang, Wei Zhang

**Affiliations:** Key Laboratory of Brain Functional Genomics (East China Normal University), Ministry of Education, Shanghai Key Laboratory of Brain Functional Genomics (East China Normal University), School of Life Science, East China Normal University, 3663 North Zhongshan Road, Shanghai, 200062 China; Department of Biochemistry and Molecular Cell Biology, Shanghai Jiao Tong University School of Medicine, 227 South Chongqing Road, Shanghai, 20025 China

**Keywords:** ADAMTS-18, Breast cancer, Knockout mice

## Abstract

**Background:**

In this study, we aimed to identify a novel extracellular proteinase ADAMTS-18 that could be a potential tumor suppressor gene.

**Results:**

We successfully constructed *Adamts-18* knockout mice with BALB / c background. RT-PCR analysis showed syngeneic mammary tumor cell line 4 T1 *per se* has weakly endogenous ADAMTS-18 expression. Orthotopic inoculation of 4 T1 cells within the mammary fat pad of host mice, we found no significant difference in tumor growth and metastasis between *Adamts-18* knockout mice and widetype control.

**Conclusions:**

We did not confirm that ADAMTS-18 in the host tissues is relevant for breast tumor progress in a murine 4 T1 breast cancer model.

## Background

Matrix metalloproteinases are traditionally linked to tumor dissemination through their ability to degrade most extracellular matrix (ECM) components, thus facilitating tumor cell invasion and metastasis [1]. However, recent studies suggest that some metalloproteinases contribute to inhibit tumorigenesis [2]. ADAMTS-18 (a disintegrin and metalloproteinase with thrombospondin motif 18), as yet no known function or substrate, is a member of ADAMTS family of secreted proteinases. It has been shown that ADAMTS-18 gene was inactivation in many carcinomas especially breast tumors and, therefore, is regarded as a novel functional tumor suppressor [3]. We previously reported that the C-terminal ADAMTS-18 fragment induces oxidative platelet fragmentation, dissolves platelet aggregates, and protects against carotid artery occlusion and cerebral stroke [4]. To further study the role of ADAMTS-18 *in vivo*, we generated *Adamts-18* knockout mice (unpublished data). The aim of this study was to investigate the effect of ADAMTS-18 on tumorigenesis in a murine 4 T1 breast cancer model.

## Methods

### Animal studies

In our laboratory *Adamts-18* heterozygote mice (*Adamts-18*^+/-^) with C57BL6/129SV were developed (unpublished data). They backcrossed to wild-type BALB/c mice for 7 generations to obtain BALB/c background *Adamts-18*^+/-^ mice. *Adamts-18*^+/-^ mice with BALB/c background are intercrossed to generate *Adamts-18* knockout mice (*Adamts-18*^-/-^) and wildtype control (*Adamts-18*^+/+^) for further experiment. Syngeneic mammary tumor cell line 4 T1 cells were orthotopically implanted within the mammary fat pad of females aged 8–10 weeks. Cells implantation in the mammary fat pad was achieved using a (1/2)- inch 26 gauge needle and gentle pressure during delivery of 100 μl cells. Mice were watched daily to monitor the growth of the tumor and once a substantial size of tumor mass was found, the mice were sacrificed and the tumor mass were collected and weighted. The surface metastatic nodules on the lungs and other organs were counted, and then histologic analyses were performed. All procedures in animal experiments were approved by the Institutional Animal Care and Use Committee of East China Normal University (ECNU). All the mice used in this study are female and maintained in a specific pathogen-free facility at ECNU.

### Western blotting

Proteins from the brain, kidney, liver or xenograft tumors of both mice were separated on a 12 % SDS–PAGE under reducing conditions and then transferred onto a polyvinylidene difluoride (PVDF) membrane. The membrane was blocked in blocking buffer (PBS, 0.5 % Tween-20, and 5 % non-fat dry milk powder) and then incubated with rabbit anti-ADAMTS18 IgG (sc-68416, Santa Cruz Biotechnology, Inc.) for 1 h at RT. After washing, the membrane was incubated with horseradish peroxidase (HRP)-conjugated secondary antibody for 1 h at RT. The immunoreactive bands were visualized with enhanced chemiluminescence (ECL) western blot kit.

### Semi-quantitative RT-PCR analysis

The endogenous expression level of ADAMTS-18 gene in murine 4 T1 tumor cells was detected by semi-quantitative RT-PCR. Total RNA was isolated from cultured cells using the TRIzol reagent according to the manufacturer’s protocol (Invitrogen, Carlsbad, CA, USA). Semi-quantitative RT-PCR was performed with cDNA reverse-transcribed from 1 μg of total RNA using AMV reverse transcriptase (Takara, Otsu, Japan). The cycle threshold values were normalized to the expression of the housekeeping gene β-actin. Band density was scanned and calculated. The primers are list as following: β-actin forward: 5′-ACGGCCAGGTCATCACTATTG-3′, β-actin reverse: 5′-CCTGCTTGCTGATCCACATCT; ADAMTS-18 forward (mouse specific): 5′-TGGAAAGTCACAAAATGGTCTCA-3′; ADAMTS-18 reverse (mouse specific): 5′- AACCACAATGTTTATGTCGCTTC-3′.

### Histological analysis

Tumor tissues were fixed in 10 % formaldehyde in phosphate-buffered saline (PBS), pH 7.4, dehydrated and embedded in paraffin and sectioned with a microtome. Tumor malignant degree and mitotic counts in sections were evaluated by hematoxylin and eosin (H.E) staining. Intratumoral vascular density was assessed by staining with goat anti-mouse CD31 antibody (Santa Cruz Biotechnology Inc., CA). Microvessel density (MVD) was determined by light microscopy in areas of invasive tumor containing the highest numbers of capillaries and microvessels per area. MVD was expressed as the number of microvessel per high-power field.

### Statistical analysis

*P* values were determined through the two-tailed Student’s *t* test. Data are presented as mean ± SD. Differences were considered statistically significant when *P* < 0.05.

## Results and discussion

The potential role of ADAMTS-18 in tumor genesis is first suggested by the genetic analysis [5]. It has been shown that the loss of 16q23 region is strongly associated with a variety of cancers. Since ADAMTS-18 is one of these genes located around 16q23 region, it has been studied as a candidate oncogene [3, 5–10]. Because ADAMTS-18 is often inactivated in breast tumors, we therefore develop *Adamts-18* knockout mouse with BALB/c background, and inoculate syngeneic mammary tumor cell line 4 T1 cells to evaluate the role of ADAMTS-18 in breast cancer.

**Fig. 1:**
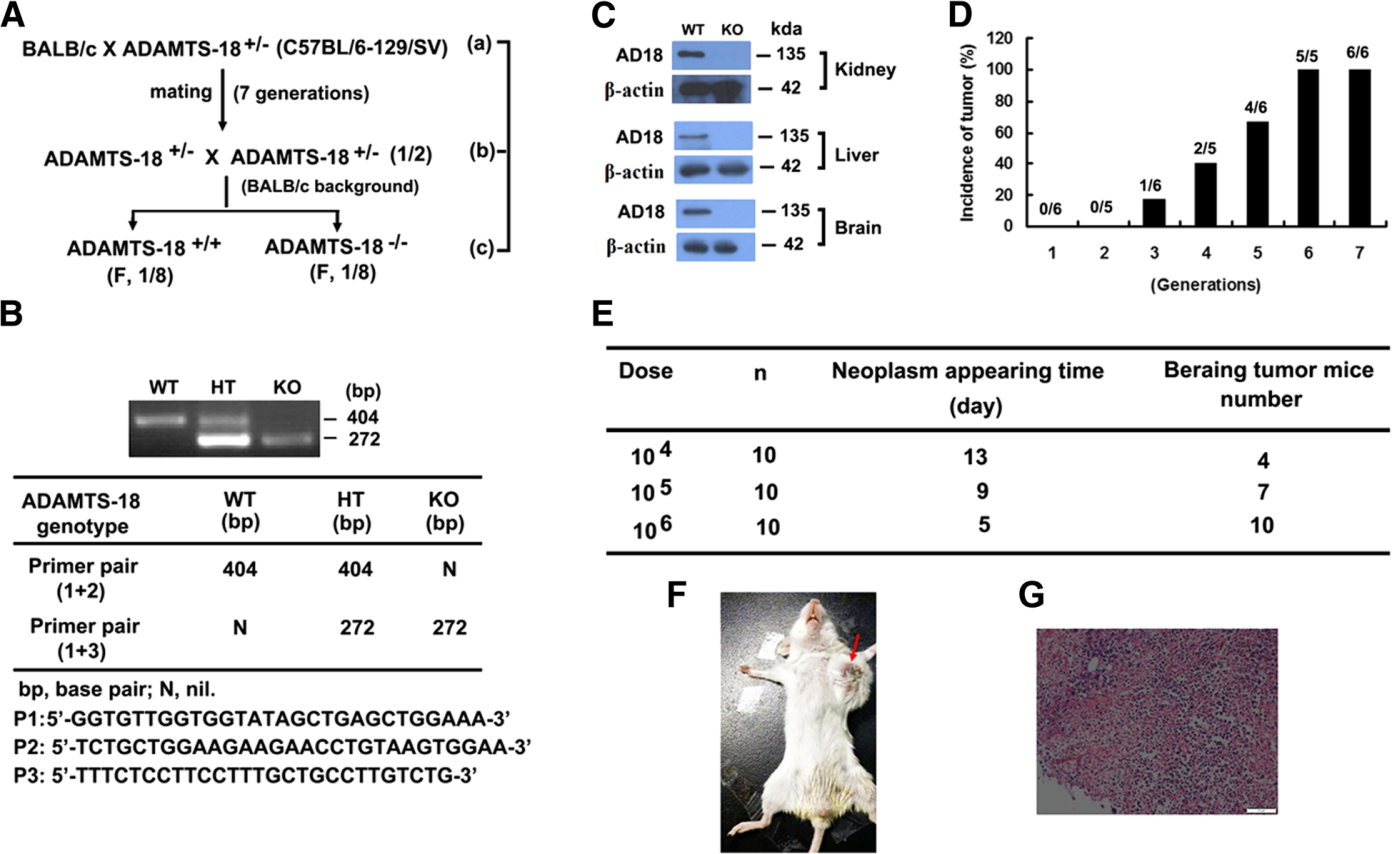
Generation and characterization of *Adamts-18* knockout mice with BALB/c background. **a** The mating strategy for producing BALB/c inbred *Adamts-18* knockout mice; (**b**) PCR genotyping of wildtype (WT), heterozygote (HT), and knockout (KO) mice; (**c**) Western blot analysis of *Adamts-18* in kidney, liver, and brain from different mice, β-actin was used as a loading control. WT, wild-type mouse; KO, knockout mouse. **d** Incidence rate of breast cancer in different generation of mice with BALB/C background; (**e**) Bearing-tumor mice number in the seventh generation of BALB/c inbred mice after orthotopically implanted within the mammary fat pad of mice with different dose of 4 T1 cells. **f** Representative photograph of breast tumor. Arrow refers to breast tumor; (**g**) Representative HE staining, bar = 50 μm

First, *Adamts-18* heterozygote mice (*Adamts-18*^+/-^) with C57BL6/129SV background were backcrossed to wild-type BALB/C mice for 7 generations to obtain *Adamts-18*^+/-^ mice with BALB/ c background. They are intercrossed to get *Adamts-18* knockout mice (*Adamts-18*^-/-^) and wildtype control (*Adamts-18*^+/+^) for further experiment (Fig. 1a-b). Immunoblot analysis for ADAMTS-18 expression in different tissues from both mice further confirmed the knockout effectiveness (Fig. 1c). The results of orthotopic inoculation of syngeneic mammary tumor cell line 4 T1 cells (10^6^/mouse) within the mammary fat pad showed that the percentage of tumor incidence reached 100 % after six and seven generations (Fig. 1d). In the seventh generation of mice, 10 of 10 mice bear tumor when the inoculation doses of 4 T1 cells were 10^6^ per mice (Fig. 1e). Representative photograph of breast tumor (Fig. 1f) and hematoxylin-eosin (HE) staining are shown (Fig. 1g).

**Fig. 2:**
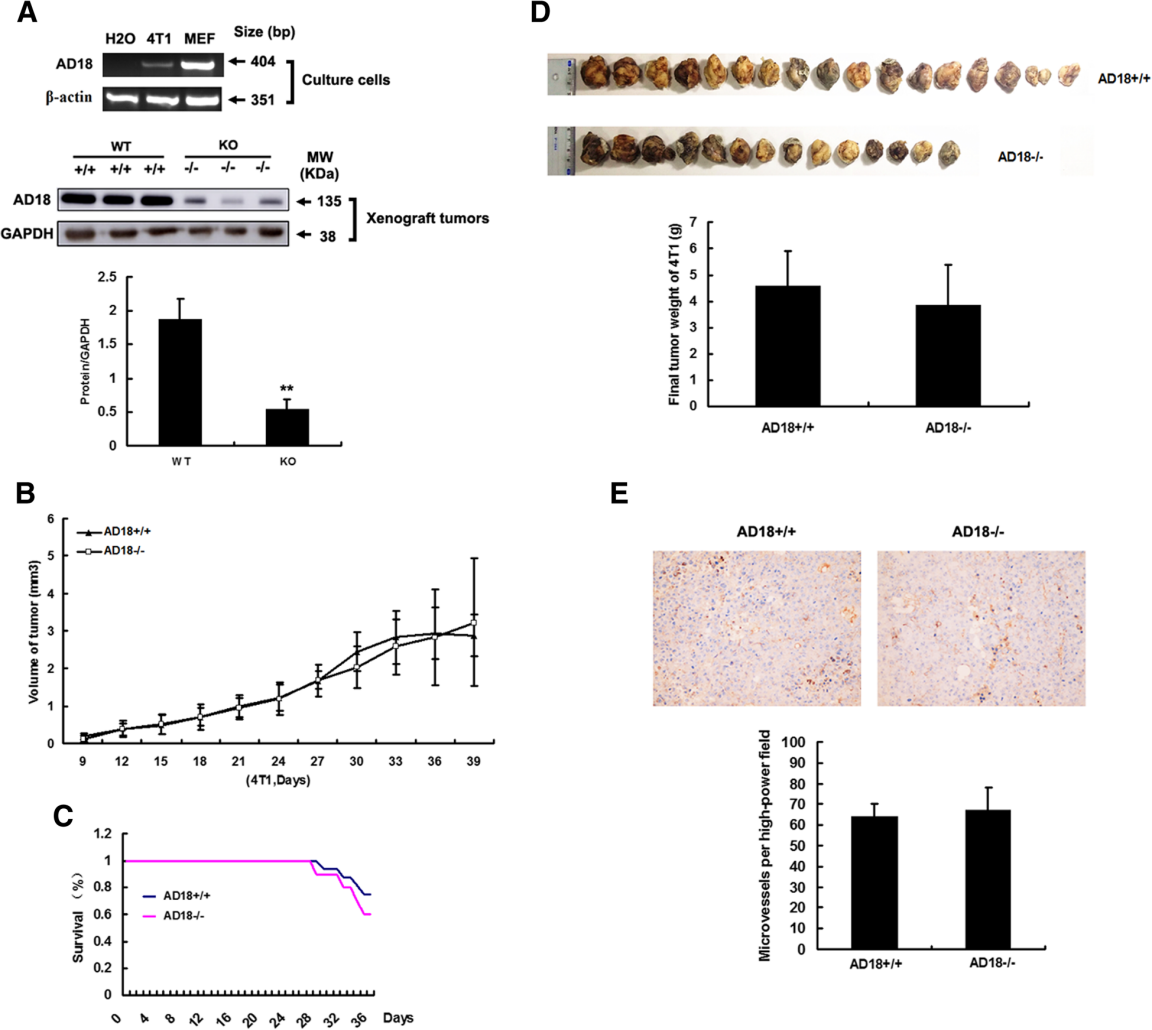
Syngeneic mammary tumor cell line 4 T1 subcutaneously transplanted growth. **a** The endogenous ADAMTS-18 expression in cultured 4 T1 cells (upper panel) or in xenograft 4 T1 tumors (lower panel). MEF, mouse embryonic fibroblast; bp, base pair; MW, molecular weight; **indicates *P* < 0.01. **b** Mice were challenged with 10^6^ 4 T1 cells, and tumor volumes were monitored for six weeks. **c** Survival rate of mice; (**d**) Final tumor weight in different genotype of mice. *Adamts-18* ^*+/+*^, wildtype mice; *Adamts-18* ^*-/-*^, knockout mice. **e** CD31 (40 × magnification) positive microvessels in each group (*n* = 5/group). Data are expressed as mean ± SD

**Fig. 3:**
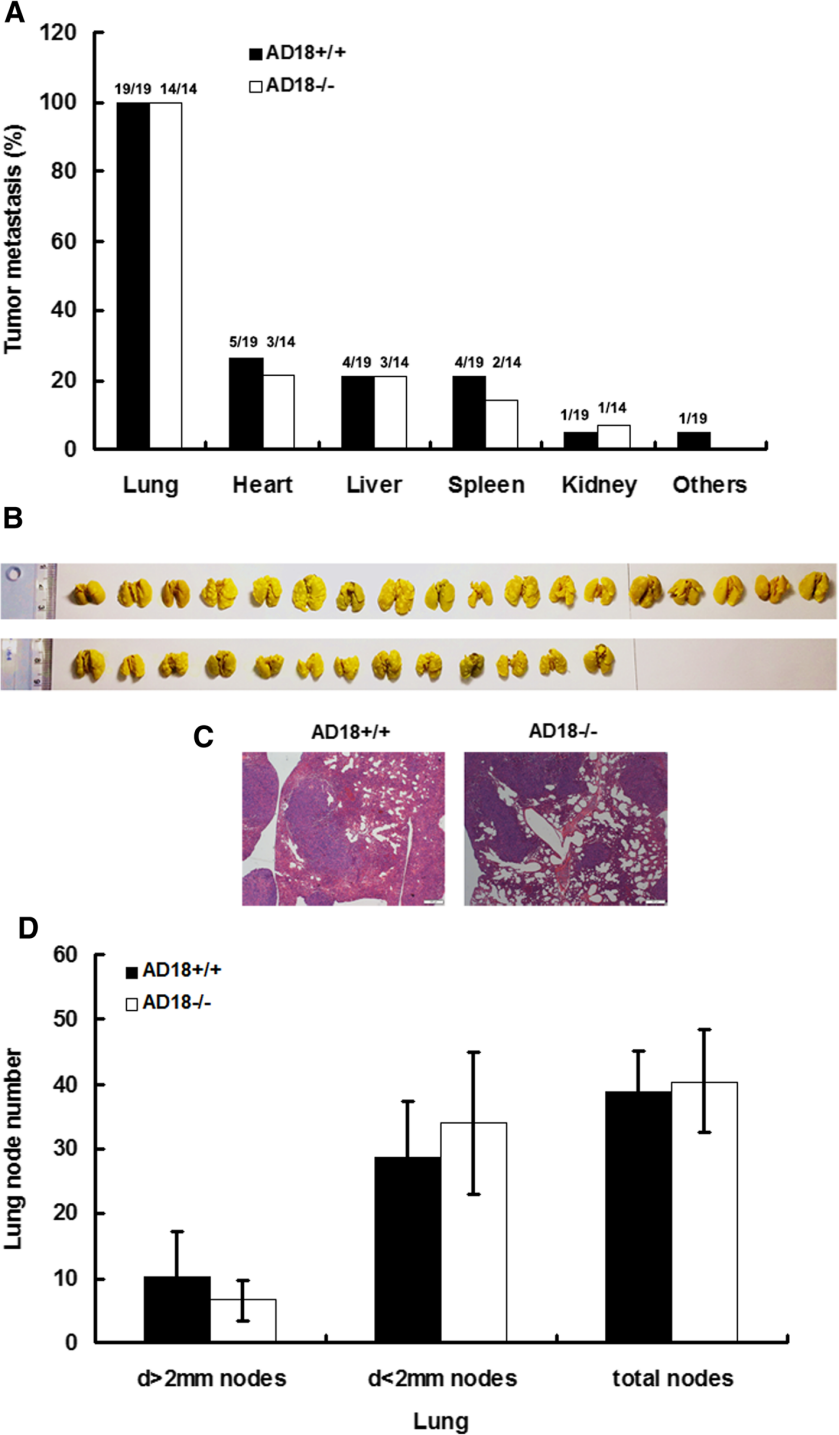
Tumor metastasis in different genotype of mice. **a** Systemic tumor metastasis; (**b**) Representative photograph of lung metastasis; (**c**) Representative HE staining of lung metastasis; (**d**) Bar graph showing statistic analysis of lung tumor nodes

The effect of tumor growth in some extent depends on tumor cell line *per se*. It has been shown that the expression of ADAMTS-18 was dramatically reduced or totally silenced in multiple human cancer cell lines, including cell lines of human breast cancer (BT549, MB231, MCF-7, and YCC-B1) due to methylation of promoter CpG Ilands (CGI) [5]. To test the expression level of ADAMTS-18 in murine 4T1 cells, RT-PCR was performed and the result showed 4T1 *per se* has weakly endogenous ADAMTS-18 expression (Fig. 2a, upper panel). In this regard, 4T1-knockout ADAMTS-18 cell line is needed in future studies. We then injected 4T1 cells to both *Adamts-18* knockout mice (*Adamts-18*^-/-^) and wildtype control (*Adamts-18*^+/+^) mice. The result of Western blotting showed that the *Adamts-18* expression is ~3.6 fold higher in xenograft tumors of WT mice than in KO mice (*n* = 3, **P < 0.01) (Fig. 2a, lower panel). However, we found no significant differences in tumor growth, survival rate of mice, tumor weight between *Adamts-18*^-/-^ and *Adamts-18*^+/+^ mice (Fig. 2b-d). There was no significant difference in tumor malignant degree and mitotic counts between *Adamts-18*^+/+^ and *Adamts-18*^-/-^ mice [mitotic counts, WT *vs.* KO, 10.75 ± 2.1 *vs.* 11 ± 1.4 per high power field (HFP), *n* = 8/group, *P* = 0.826). Moreover, microvessel density (MVD) had no significant difference between *Adamts-18*^+/+^ and *Adamts-18*^-/-^ mice (MVD, WT *vs.* KO, 64 ± 6 *vs.* 67 ± 11 per high power field (HFP), *n* = 5/group, *P* = 0.947) (Fig. 2e). There is no significant difference in systemic metastasis (Fig. 3a), especially lung metastasis (Fig. 3b-d) between *Adamts-18*^+/+^ and *Adamts-18*^-/-^ mice. These results indicated that ADAMTS-18 in host tissues (e.g. stroma components, endothelium, tumor-associated fibroblasts, and immune cells etc) likely exerts little effect on breast tumor progress. However, further studies are needed to assess the effect of ADAMTS-18 on other tumor types (e.g. melanoma, spontaneous cancers, etc) or its synergy effect with other tumor-related genes.

Many ADAMTSs have proved to be anti-angiogenic through their metalloproteinase-dependent and –independent activities, which contribute to inhibit cancer development and progression. ADAMTS-1 displays both types of mechanism [11]. It induces the generation of anti-angiogenic fragments by cleaving matrix-bound thrombospondins-1 and -2 [12], and also sequesters angiogenic factors such as the basic fibroblast growth factor (bFGF) and vascular endothelial growth 1(VEGF165) [13, 14]. ADAMTS-9 also demonstrates metalloproteinase-dependent and –independent activities, but the mechanism remains unclear [15]. Other ADAMTSs, including ADAMTS-2, -4, -5, and -12, displays metalloproteinase-independent inhibition of neovascularization, which is closely related to their thrombospondin type 1 sequence repeat (TSR) domain [16–19]. The peptides (termed Adamtsostatins) corresponding to the TSRs of some family members have anti-angiogenic properties, indicating that this may be a general activity of ADAMTSs. In this study, we didn’t find difference in microvessel densities of tumors between *Adamts-18*^+/+^ and *Adamts-18*^-/-^ mice. Nevertheless, more sophisticated assays for angiogenesis such as wound healing, tube formation, and ischemia assays are needed in future studies.

## Conclusions

The current data suggest that the murine 4 T1 breast tumor cell *per se* has weakly endogenous ADAMTS-18 expression, and ADAMTS-18 in the host mouse exerts little effect on breast tumor progression in murine 4 T1 breast cancer model.

## Abbreviations

ADAMTS-18: a disintegrin and metalloproteinase with thrombospondin motif 18
ECM: extracellular matrix
HE: hematoxylin-eosin
